# Developmental genetic underpinnings of a symbiosis-associated organ in the fungus-farming ambrosia beetle *Euwallacea validus*

**DOI:** 10.1038/s41598-023-40296-1

**Published:** 2023-08-28

**Authors:** Ellie J. Spahr, Fady Wasef, Matt T. Kasson, Teiya Kijimoto

**Affiliations:** Division of Plant and Soil Sciences, West Virginia University, Morgantown, WV USA

**Keywords:** Evolutionary developmental biology, Morphogenesis

## Abstract

Mutualistic interactions between organisms often mediate the innovation of traits essential to maintain the relationship. Yet our understanding of these interactions has been stymied due to various hurdles in studying the genetics of non-model animals. To understand the genetic mechanisms by which such traits develop, we examined the function of genes *breathless* (*btl*), *trachealess* (*trh*), and *doublesex* in the development of a novel fungus-carrying organ (mycangium) that facilitates an obligate relationship between fungus-farming ambrosia beetles and specific fungal partners. Gene knockdown by RNA interference and subsequent micro-computed tomography visualization suggest *btl* and *trh* are required for initiation of mycangia and that tubulogenesis may have been co-opted for early mycangial development.

## Introduction

Evolutionary developmental biology seeks to address fundamental questions surrounding the mechanisms underlying the morphological innovation and evolution towards increasing structural complexity within the body plan. Co-option allows for the reuse of core developmental genes in a novel developmental context such as the integration of insect appendage genes in the evolution of butterfly eye spots^1^. Among many interesting examples, novel traits that can assist in partitioning a microbial symbiont within the animal host body are of particular relevance. The Hawaiian bobtail squid (*Euprymna scolopes*) and its symbiont *Vibrio fischeri* are one such consortium where identification of co-opted gene targets within the squid’s light organ has helped decipher the identity and evolutionary origins of this structure^2^.

Ambrosia beetles, which comprise more than 3500 species among true weevils (Curclionidae), have independently formed obligate nutritional partnerships with fungi over a dozen times^3^. Their ability to vector fungal symbionts within specialized organs, called mycangia, has led to many investigations on morphology and function. However, outside phylogenetic research, few studies provide molecular genetic resources^4,5^, and the genetic and developmental mechanisms underlying repeated evolution of mycangia is still poorly understood. Mycangia can be found in a variety of body segments with varying degrees of complexity; some mycangia present as shallow pits, while others are more complicated invaginations, pockets, or tubules^3,6^. This structural adaptation is associated with a shift in both larval nutritional composition and the natal gallery structure within woody environments when compared to their phloem feeding bark beetle relatives^7^. As such, xylomycetophagy is essential to the ecological niche defined by ambrosia beetles and their fungal symbioses^7^. This evolution to xylomycetophagy has occurred between a great diversity of fungal partners^6^; diverse nutritional associates leading towards the same structural and functional end point suggests these close nutritional relationships may drive specialized development within the host insect.

The relatively derived trait of fungal culturing has facilitated the beetles’ ability to thrive in nutritionally depleted woody habitats by enabling them to utilize lignocellulose as a valuable resource. While their wood-boring behavior primarily targets stressed or dying trees, the extensive attacks on host trees can exacerbate their decline. In today’s globalized world, the introduction of exotic ambrosia beetle species increases the likelihood of novel interactions with native trees, thereby amplifying the potential for new host associations to arise. Consequently, ambrosia beetles have emerged as a significant subject within the fields of forest pathology and entomology. Additionally, the diversity in mycangia morphology and symbiotic relationships with fungal partners renders them highly intriguing from an evo-devo perspective. Here we used the ambrosia beetle species *Euwallacea validus* to study the genetic underpinnings of mycangia development. This species, which was introduced to the US in the late 1900’s, is widely available in the Northeastern United States. Adult female *E. validus* vector their primary nutritional symbiont, *Fusarium oligoseptatum*, in presumably a pair of preoral mycangia that are medially located within the head behind the mandibles. Previous research resolving internal organization of mycangia identified a second pair of small pouches within the head, inferior to the primary mycangia and located laterally near each eye, but did not extend to functional characterization^8^. *Euwallacea* species are also known to vector secondary symbionts (such as *Raffaelea subfusca* and *Graphium sp*.) which are theorized to play alternate roles within natal galleries across development^9^. Other ambrosia beetle species have been found to not only develop multiple mycangia but may also segregate different symbionts in separate pouches^6^. Resolution surrounding the *Euwallacea* genus leaves many questions unanswered regarding peripheral partnerships but has shown a high-fidelity relationship between these beetles and *Fusarium*. Examination and culture-based sampling of 250 female *E. validus* resulted in recovery of fungi from 99.2% of sampled individuals indicating robust conservation of mycangia and fungal colonization in wildtype populations^10,11^.

Our previous research showed that preoral mycangia develop during the pupal stage in *Euwallacea validus*^8^. To discern early candidates that may be involved in mycangial development, we selected two genes, *trachealess* (*trh*) and *breathless* (*btl*), based on the structural features of preoral mycangia and their repeated deployment during tubulogenesis across insect development. The transcription factor Trachealess (Trh) has been shown to regulate the expression of fibroblast growth factor receptor Breathless (Btl) during the initiation of tubulogenesis – a developmental process used in both salivary gland and tracheal morphogenesis in *Drosophila* embryogenesis^12^. Although mycangia are functional only in females^10^, our previous research suggested males develop protomycangia, a potential equivalent of mycangia that are reduced in size with likely no function in carrying fungal propagules^13^. To explore any sex-specific regulation involved in the development of these sexually dimorphic structures, we also examined the function of gene *doublesex* (*dsx*).

We developed an RNA interference-mediated gene knockdown system by which we examine the function of candidate genes in the development of mycangia. To measure gene expression level during mycangia development, we utilized quantitative RT-PCR across the female beetle life cycle. To observe the phenotype of RNAi animals, we utilized microcomputed tomography (µCT)^8^. This research provides the first report on genetic contributions in the development of mycangia, a beetle organ critical to maintaining the symbiotic relationship.

## Results

### Quantitative RT-PCR results suggest sustained expression of tubulogenesis genes

**Figure 1:**
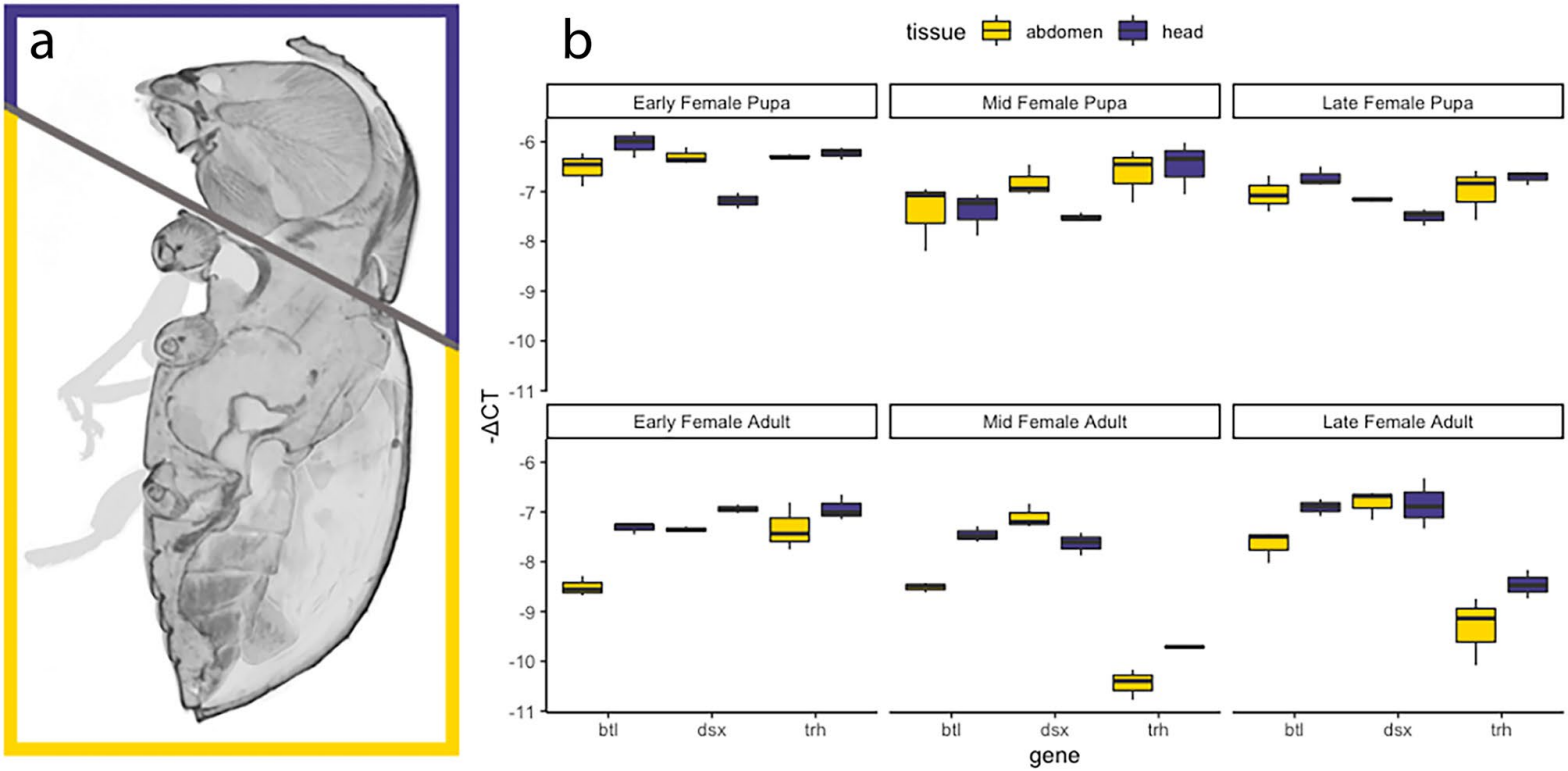
qPCR in female *E. validus* beetles across development and between head and abdominal tissues. (**a**) *E. validus* tissue dissections collected for head (purple) and abdominal (yellow) tissue samples analyzed in qRT-PCR. (**b**) −ΔCt value for head and abdominal tissues across lifestage. Results between tissues were not significantly different (see Supplemental Table S1 for *p*-values).

**Figure 2:**
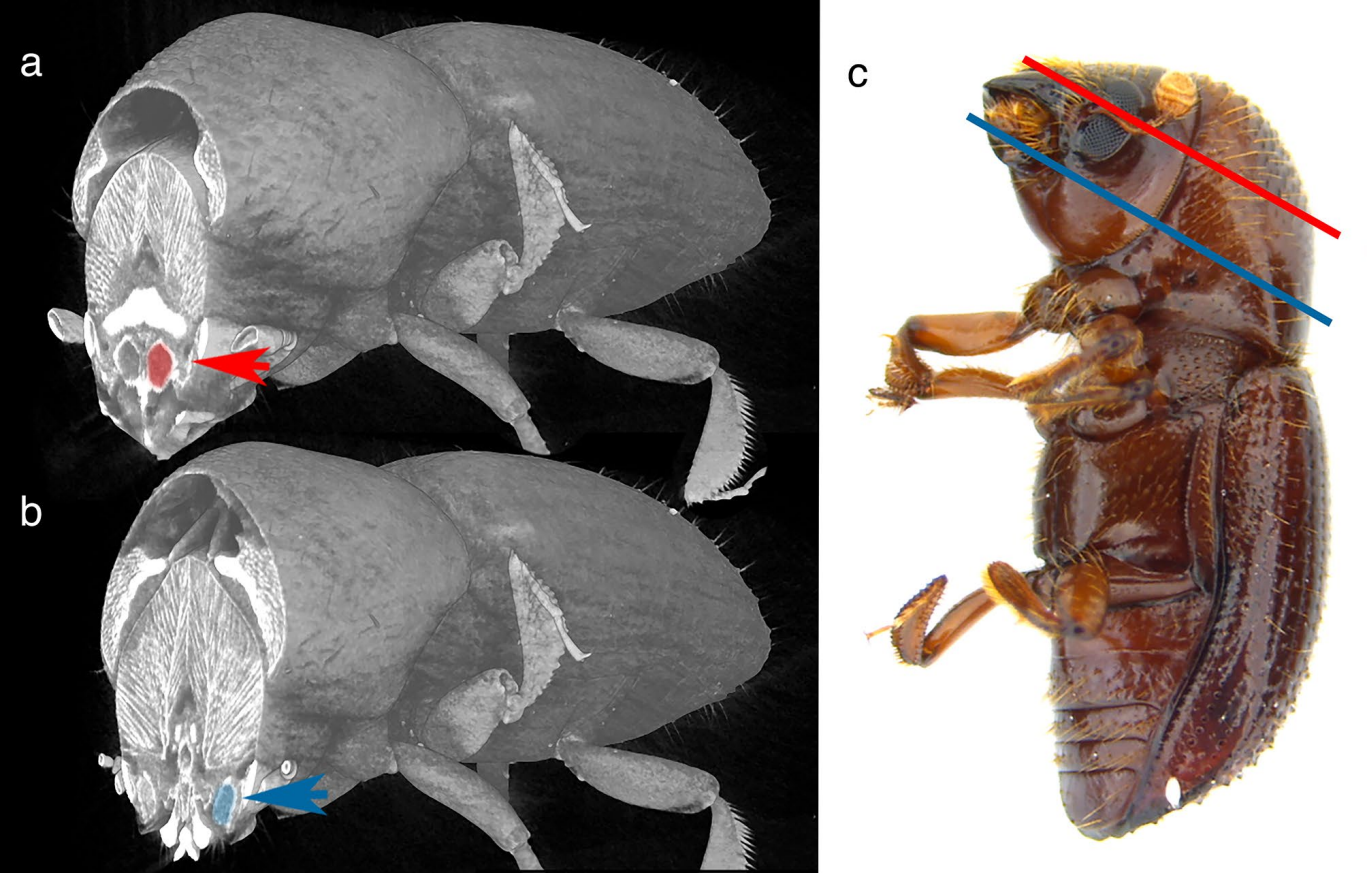
The morphology of mycangia in *E. validus*. (**a**) Transverse µCT image showing the major, medial pair of mycangia. One of mycangia is shaded in red (red arrowhead). (**b**) Transverse µCT image showing the minor (inferior) pair of mycangia-like pouches. One of the structures is shaded in blue (blue arrowhead). (**c**) An external image of a premature adult female correlates external morphology to plane of digital cross-section. Lines indicate the transverse section plane in a (red) and b (blue).

To examine whether candidate genes are expressed in mycangia and the degree of differential expression between body segments, we conducted quantitative RT-PCR. Although mycangia in other species have been dissected from thoracic segments (*Xylosandrus germanus*^5^), in *E. validus*, the mycangia develop inside the hard head capsule. Further, our previous study suggested their mycangia to be complex therefore we utilized the whole head in this study to avoid any potential bias during the sampling process (Fig. 1a). Overall, our results indicated all candidate genes were expressed in heads and abdomens in all stages tested (Fig. 2)*.* However, our statistical analyses did not support a difference in the average candidate gene expression levels between head (including mycangia) and abdomen.

### RNAi-mediated gene knockdown induced malformation of mycangia

**Figure 3:**
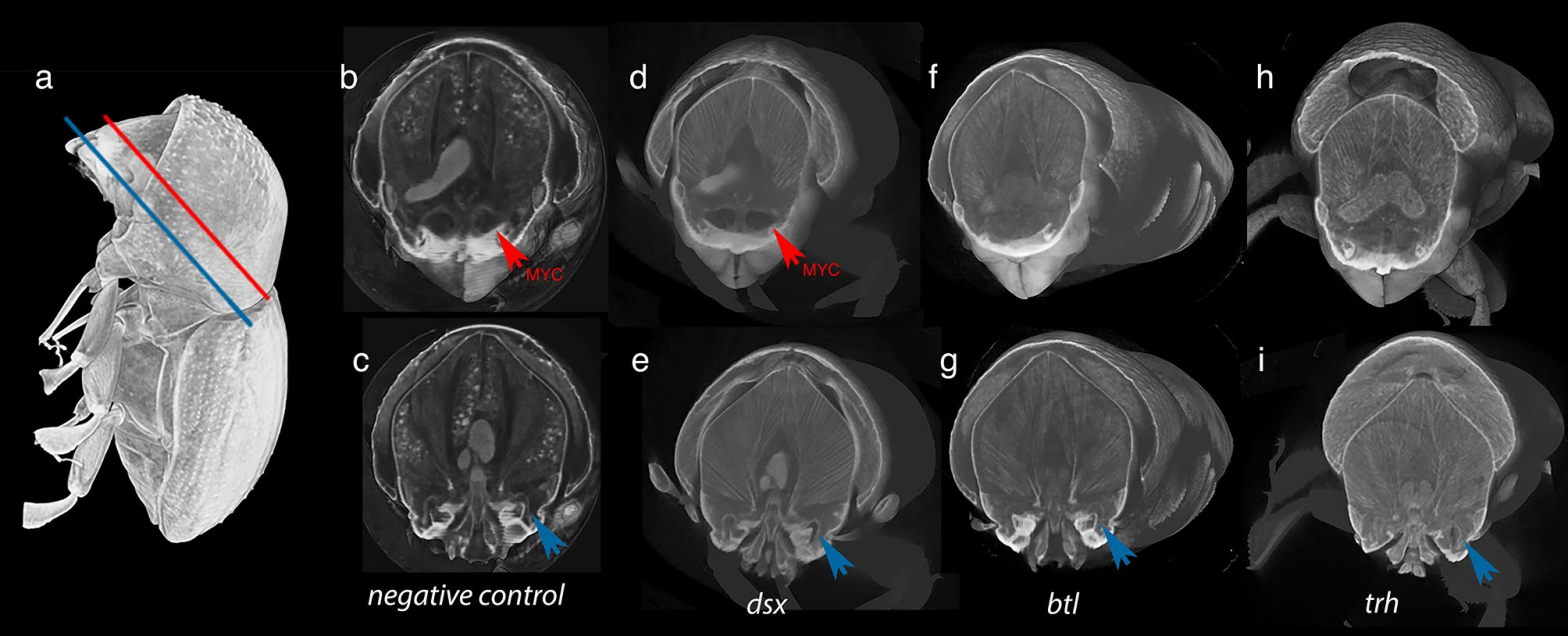
Representative RNA Interference phenotypes. (**a**) Planes of virtual dissection for superior (red line corresponding to results **b**, **d**, **f**, **h**) and inferior (blue line corresponding to results **c**, **e**, **g**, **i**) cross-sections. Micro-CT scans show intact superior mycangia (red arrow) in the control (**b**) and *dsx*-dsRNA treated insects but no equivalent structures within *btl*-dsRNA (**f**) or *trh*-dsRNA (**h**) treated animals. Inferior mycangia-like spaces (bottom; blue arrow) are visible across negative control (**c**), *dsx*-dsRNA (**e**), *btl*-dsRNA (**g**), and *trh*-dsRNA (**i**) treatments. Inferior mycangia were not disrupted in any treatment.

**Figure 4:**
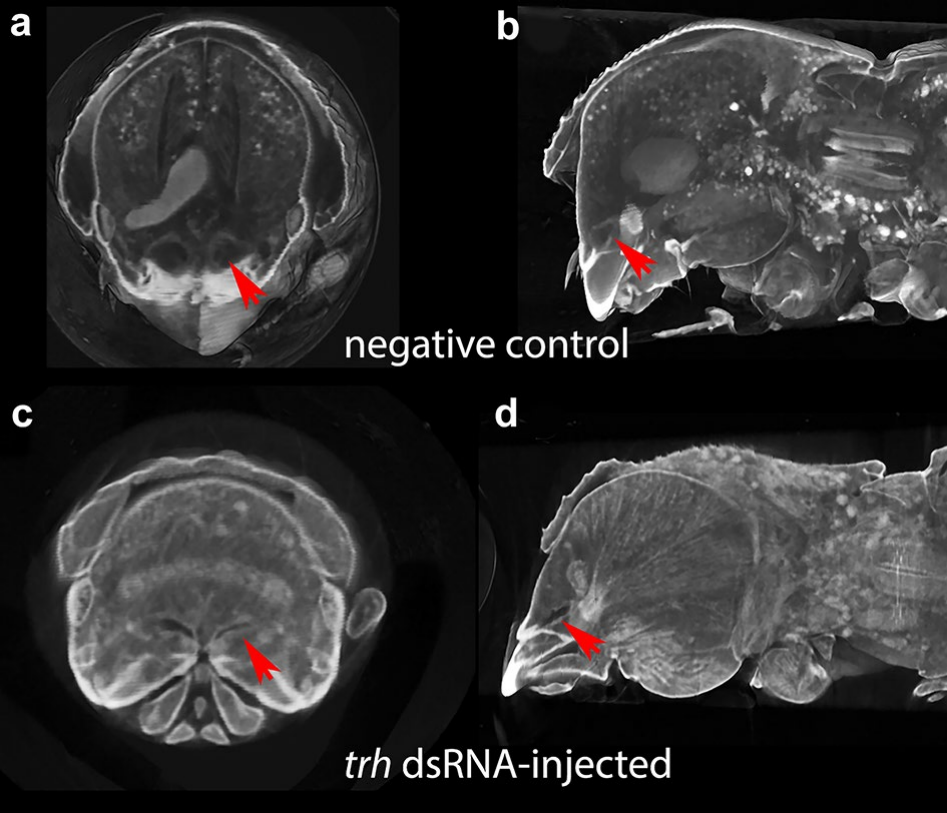
Representative phenotype of intact but reduced *E. validus* mycangia following *trh*-dsRNA treatment. Mycangia in negative control (**a** and **b**) is not affected by treatment while *trh*-dsRNA treated individual (**c** and **d**) exhibits irregular morphology. Red arrows denote mycangia in each digital cross-section.

Our previous morphological study suggested that *E. validus* harbor complex, potentially dual-paired mycangia, a major pair situated in the more anterior and medial head and a minor pair in the inferior (ventral) lateral part of the head (Fig. 2)^8^. To examine the function of candidate genes, we established a protocol of larval RNAi. Overall, RNAi treatment affected the development of the major pair of mycangia, but minor mycangia may not be affected in their morphology (Fig. 3). In *btl*-dsRNA treated animals, mycangia were absent in 3 of 6 individuals. For *trh*-dsRNA injected animals, mycangia were absent in 3 of 6 animals. Of those animals that developed mycangia within *btl*-dsRNA and *trh*-dsRNA treatments, overall volume appeared reduced in 2 individuals (Fig. 4). In cases where the structure was absent, there was no distinct boundary or empty gap where mycangia would have developed (Fig. 3f and h); mycangial membranes were not detectable at any angle or by modification of contrast, despite the clear presence of other stained soft features within the head such as muscle fibers. Micro-CT results suggested that females injected with *dsx-*dsRNA during the larval stage all developed mycangia at adulthood (7 of 7 females, Fig. 3b and c). Negative control was not distinguishable from non-treated wild type animals.

## Discussion

Due to specific relationships, the evolution of symbiosis can be associated with the development of unique features in both hosts and symbionts^2,14,15^. Here, we utilized an ambrosia beetle species *E. validus* to study the developmental genetic basis of specialized, symbiosis-associated organs known as mycangia. We targeted two developmental processes, tubulogenesis and sex determination based on morphological observation and past research that suggested mycangia are functional only in females^8,10,12^. Results from quantitative RT-PCR and RNAi suggested target genes are at least partially responsible for the development of mycangia. Below, we further discuss the potential significance of those target genes.

If any gene plays a critical role in organogenesis, gene expression is expected to be upregulated during the relevant developmental time period, however, our quantitative RT-PCR results indicated no significant increase in the expression of *trh* and *btl* between life stages. This suggests that these genes are functionally expressed throughout the beetle life cycle. Indeed, in *Drosophila*, *trh* expression is constitutively maintained in tracheal cells even after tracheal development has been initiated^16–18^. Alternatively, potential variability in the stage determination might have affected the high variability of ΔCT values (see Supplemental Table S1 for *p*-values). We staged pupae and adults by their body color (sign of sclerotization), however staging animals by the time (e.g., days after molt) may increase the resolution of gene expression pattern analyses.

Although the effect was up to 50% (3 of 6 animals showed the same mycangia-less phenotype in both *trh*- and *btl*-dsRNA treatment), our RNAi method generated a subset of animals with notably malformed mycangia in treatments associated with tubulogenesis genes. In addition, some animals indicated reduced size of mycangia after the treatment. Together with the qPCR results, we propose that genes *trh* and *btl* are responsible for the initiation of mycangia development in *E. validus*. The success rate of larval RNAi treatment may vary due to the target gene function or differences in larval maturity at time of injection. As discussed above, the function of these genes can be critical to the survival of animals therefore early knockdown may lead to early mortality and a reduced proportion of surviving, treated adults.

The integration of *trh* and *btl* in mycangial initiation would suggest tubulogenesis has been co-opted in the evolution of preoral mycangia in *E. validus* and, unlike legume nodulation, beetles may not require the fungal symbiont to initiate the development of mycangia. Indeed, the independent structural initiation is supported by observations of aposymbiotic development in the ambrosia beetle genus *Xylosandrus*^19^. It is worth noting that these genes may be involved in the development of only the major pair of mycangia in this species, which also suggests the independent origin of major and minor mycangia.

Across the larger ambrosia beetle taxonomy, sex-specificity of traits varies between beetle clades. In *Euwallacea*, mycangia were previously thought to be female-specific and as such investigations focused on a female model^13,20,21^. *dsx*-dsRNA injected insects showed intact mycangia at the adult stage and mycangia were visible across all samples (7 of 7, Fig. 3b). In *dsx*-dsRNA injected beetles, the membrane remained intact suggesting *dsx* knockdown did not impair initiation or completion of mycangia. This suggests that the morphological differences we have observed previously between sexes^8^ might not be as simple as beetle horns where *dsx* regulates sex-specific horn development^22^, and, rather, may result from other mechanisms such as ploidy and the associated gene expression patterns^23,24^.

Co-option has been shown to integrate existing developmental programs in the development of symbiosis associated novelties. In legumes, a lateral root transcription factor is deployed in the root cortex to co-opt components of lateral root development for patterning nodules^14,15^. In the light organ of the Hawaiian bobtail squid, *Euprymna scolopes*, eye genes have been co-opted in the development of the light-responsive photophore^2^. That mycangia may employ portions of existing developmental pathways – such as tubulogenesis – to initiate development is congruent with other models for the evolution of novel structures. This study provides the first example of potential co-option of genes that are critical for tubulogenesis and the development of mycangia. Information gained by categorizing the novel gene regulatory network responsible for mycangial development in *E. validus* may provide a model that can be tested in future work with other species across clades of distinct evolutionary origin to determine if the same regulatory and structural genes have been co-opted to pattern similar structures.

## Methods

### Animal husbandry

Insects were collected from *Ailanthus altissima* logs and reared in the laboratory as previously described protocols^8^. Briefly, adults were mechanically extracted from split logs and then raised in sterilized agar medium that contains *A. altissima* sawdust. Larvae were transferred onto glucose yeast extract agar (GYEA) plates with fully colonized *Fusarium oligoseptatum* lawns as a food source. Pupae were transferred into artificial galleries within sawdust water agar plates and monitored until they completed their final molt.

### Total RNA extraction and sequencing

Animals were flash frozen in LN_2_, and ground with mortar and pestle for total RNA extraction using the QIAgen RNeasy Plus Micro RNA extraction kit. Reverse Transcription was conducted using SuperScript IV Kit to generate cDNA libraries from total RNA. mRNA sequences were retrieved for genes of interest (*dsx, btl, trh*) from the NCBI gene database to design degenerate primers from amino acid consensus sequences. Partial gene sequences were deposited in NCBI GenBank (OP948674-8).

### dsRNA synthesis and injection

Preparation of dsRNA as described by Kijimoto et al.^22^. Primers for each candidate gene (*doublesex* (*dsx*) (F: 5′-GAACTTGTTGTCCGTGCCTC-3′, R: 5′-GTACTAGTTGCCGGTGCAGC-3′), *breathless* (*btl)* (F: 5′-CTTCCAGCAAACGCAGTG-3′, R: 5′-TGCGATCATTTTCTCCCGT-3′), and *trachealess* (*trh*) (F: 5′-AGGAAGTGGTTCATAAGAAA-3′, R: 5′-GTATTAAAACAACCCTATACC-3′)) were designed to amplify ~ 150 bp regions of each gene. 85–125 ng of dsRNA was delivered to each larva via 13.8 nL injections using a Nanoject Micropipette and pulled glass needles. Larvae were injected in the first prothoracic segment, directly behind the head capsule. Injected larvae were placed on GYEA plates with fully colonized *F. oligoseptatum* lawns to recover and mature.

### Micro-computed tomography

Scanning and staining was completed as described by Spahr et al.^8^. External morphology photographed prior to staining using a Leica Stereomicroscope. A total of 19 insects (6 *trh*-RNAi, 6 *btl*-RNAi, and 7 *dsx*-RNAi) were scanned and analyzed; samples were omitted from analysis if staining occlusion, scan quality, or mechanical damage prevented observation at the relevant spaces within the head.

### Quantitative RT-PCR

Female *E. validus* were collected at the pupal and adult stage and separated by maturity based on external features. Fifteen insects were collected for each of the six lifestages. Five insects were aggregated into a single biological sample, with three biological replicates processed for each life stage. Insects were dissected into head and abdominal sections (Fig. 1A) before immediate flash freezing in LN_2_ and storage at -80°C. RNA was extracted using Qiagen’s RNeasy Plus Micro kit and synthesized to cDNA using ThermoFisher SuperScript IV VILO Master Mix; reverse transcription was conducted using 200 ng RNA. Sample purity was assessed through Nanodrop absorption ratios and gel electrophoresis. Potential gDNA contamination was eliminated through gDNA eliminator columns during RNA extraction and ezDNase treatment during reverse transcription. qPCR was conducted using the PowerTrack SYBR Green protocol with the QuantStudio 3 (Applied Biosystems) Real Time PCR machine Comparative Ct analysis program (QuantStudio v.1.5.0) using multiple endogenous reporters and ROX passive reference signal. Expression of target genes (*dsx, btl, trh*) was standardized to two endogenous reference genes, *ribosomal protein subunit 3* (*rps3*) and *elongation factor 1 alpha* (*ef1a*) (Fig. 1B). qPCR reactions were prepared at a 20 μL reaction volume as according to PowerTrack SYBR Green protocol with 1 μL of cDNA per reaction (corresponding to either 5 ng or 10 ng equivalent starting RNA by sex). Primer sets for target genes *doublesex* (*dsx*) (F: 5′-GAACTTGTTGTCCGTGCCTC-3′, R: 5′-GTACTAGTTGCCGGTGCAGC-3′), *breathless* (*btl)* (F: 5’-CTTCCAGCAAACGCAGTG-3’, R: 5’-TGCGATCATTTTCTCCCGT-3′), and *trachealess* (*trh*) (F: 5′-ACTCCAAAAATGCCGAAGAA-3′, R: 5′-TTTCTGGGTCATTACTGCTG-3′) and endogenous reporters *ribosomal protein subunit 3* (*rps3*) (F: 5′-ATTGTGCGCTATTGCCCAAG-3′, R: 5′-TGTCCTCTCAATTTGCCCGA-3′) and *elongation factor 1 alpha* (*ef1a*) (F: 5′-ACCATCATTGATGCCCCTGG-5′, R: 5′-AGCATGCTCTCTGGTCTGTC-3′) were developed to amplify 150 bases at an annealing temperature of 54 °C and were included in each reaction at a final concentration of 400 nM. Primer robustness and specificity was tested in PCR dilution series prior to analysis. Quantitative RT-PCR was conducted with the following cycle parameters: 2 min at 95 °C, followed by 40 cycles of 95 °C for 15 s, 54 °C for 15 s, and 72 °C for 30 s, then 95 °C for 15 s. A melt curve was conducted from 54 to 95°C with camera capture during stage 3. Biological replicates were assessed in triplicate, with three technical replicates per sample. Gene expression was standardized to the endogenous reporters for ΔCt values and normalized to abdominal tissue for each lifestage in the calculation of ΔΔCt values. A Wilcoxon Signed Rank test was employed to test for significance of ΔCt values between tissues by gene and stage.

### Supplementary Information

Supplementary Information.

## Data Availability

Associated sequence data can be found in the NCBI GenBank (https://www.ncbi.nlm.nih.gov/genbank/) under Accessions [OP948674 through OP948678]. The sequence datasets generated during this study are available in Supplementary material.
