# Supplementary material for: Developmental genetic underpinnings of a symbiosis-associated organ in the fungus-farming ambrosia beetle Euwallacea validus
Source: Sci Rep. 2023 Aug 28;13:14014. doi: 10.1038/s41598-023-40296-1 (PMC10462615; doi:10.1038/s41598-023-40296-1)
Supplement: Supplementary file 1 — Supplementary Information. [file 41598_2023_40296_MOESM1_ESM.pdf]

Supplemental Table S1: Statistical significance between head and abdomen ΔCT values across suggest no statistical differences in gene expression. The Wilcoxon Signed Rank test was used to test the significance between head (n=3) and abdomen (n=3) ΔCT values of each candidate gene at each tested life stage. Non-parametric testing was used due to small sample size and no assumption of normality.

**Life Stage**	***dsx***	***btl***	***trh***
Early Female Pupa	P-value = 0.2	P-value = 0.2	P-value = 0.7
Mid Female Pupa	P-value = 0.1	P-value = 1	P-value = 0.7
Late Female Pupa	P-value = 0.1	P-value = 0.4	P-value = 1
Early Female Adult	P-value = 0.1	P-value = 0.1	P-value = 0.4
Mid Female Adult	P-value = 0.1	P-value = 0.1	P-value = 0.1
Late Female Adult	P-value = 1	P-value = 0.1	P-value = 0.1

Supplemental Data: Sequence data from genes studied in this research.
>Seq1 [organism=Euwallacea validus] breathless mRNA, partial CDS
TGAAGCCGGCTGGAAACATGGTGCAGCTGAAATGCAGGGCTGGGGAAACCCTACTCCGAATATAACCTGGCT
CAAAGATGGCAAAACACCGTATAGAAATTTCGGAGAGTACAAAATAAACCACTGGTCTTTCACCTTAGAAGATTT
GGTTACCGATGATAAAGGCAATTACACCTGCATAGTTTGCAATGAGGCGGGATGTATCGATTTCACGTATCGATT
GGATGTTGTTGAGAGATATCACAATAGCCCAATTCTGCTTAAAAGCCCTGAGAACATAGTTGCCTTGGTTGGCAG
CAACGCGTCGTTTAAATGTAATTTTCTTAGCGATTTACATCCCTATATCACCTGGGTCAAGATGGAAAATAATTCG
AACTCGAACTCCACTGAACAGATCCAAGGTCAAGTTCTTAATTCCAACGTGTTGAATCCGCAAATACTGGAAATTT
CCAACGTTACTTACGAGGATGAAGGGTGGTACGCTTGCATAGCTGCTAACAGCCTTGGCCAAACGACTGCAAAG
GCATACCTCAAAGTAGTGGATGTTCTTCCAGCAAACGCAGTGGAACCGAAGCAAATTACTGTAAGAATCGGCCTA
ATAATTGCTGCAGTCGCTGGCCTGGTATTTCTGCTCCTCGCCTACATTTGCCTTTACTATAAGCAGAAAATCCGAA
GGGAAAAACGGGAGAAAATGATCGCAGTG

>Seq2 [organism=Euwallacea validus] doublesex mRNA, partial CDS
TCGGAGAGGCACAGGGTTATGGCCGTGCAGACTGCCCTTAGGAGGGCCCAGGCCCAAGACGAGGCGATGTTA
AAAAACGGAAATTTGGACGTGTCTCGGGATATGAACTTGTTGTCCGTGCCTCAGAAGATTCCAAGTCCCATGCAA
CCTGTAAGTTTGGACTGTGATTCATCCGCCTCGTCGCAATGTTCGGATCCGCCCATACCGCCCAGTCGGAGAGC
TTCTCCGGTACTGGCTGCACCGGCAACTAGTACCTCCATGCCCATGGGAATTGGGAATAACGAAGTTATCTCATA
TGATTATAGAGGTCAGTCTTCCCATTATCGGCATAAATCAGTGTTAGAAGGTCACCAAAGCACAGACCTCCTAGA
AGACTGTCACAAACTGTTGGAGAAGTTCAACTATCCCTGGGAGATGATGCCTCTGGTATACACCATATTGAAGGA
TGCCCGAGCAGACCTCGAAGAAGCTTCGAGGCGAATAGATGAAGCACGTTTGGAAATTCGTGCCAAGGCAGCG
GAAGAAGCAGCAGCGCGATACTCCCGCCAGATTCAATATAGCAATTTGTACTCGTCAGCATCCGCCATTGCTGC
CCTCTATCCGCCCGTTTATCTGCCTTCGATGACGGTCTACCCCCACACGACCTCATCCCTCATGCCGCCCAGGG
TGCCGCCCATTACGTATTCATCGCCACCCTCGCCTACCTTACCTCACAACTTAACCGTACCGTTCAGACCGCGTA
GCCGGTCGGGATAGATTTAGGTGAGCTCACTTTTTTCGAAACTGTAGTACTCTGTAATATTTTGTATAATTACTCA
TTATGGTAACACGACTATTTTTAATACAATACAAAAAAAAAAA

>Seq3 [organism=Euwallacea validus] trachealess mRNA, partial CDS
GTATTACAGGCCCTTGACGGCTTTGCTCTCGCCGTAGGATCAGACGGCAGATTTCTGTACATTTCTGAGACTGTT
TCAATTTATTTAGGACTATCACAGGTTGAAATGACGGGCAGTAGCATATTTGACTACATTCATCATCAAGACCATT
CAGAAATAGCTGAACATTTAGGTTTAGGATTATCCCAGGGTCAGAGCATGGCCTCACCAGGAAGTGGTTCAgAAG
AAAGTGGTTCAACGGTGGGGACAAACAACCCTGACGTCTCTACAGTCATGTCATTAGGAACAAATCCGCCTTACA
AAGGCTTAGATAGGGCATTTTGTATTCGAATGAAATCCACACTTACCAAGAGAGGCTGTCATTTCAAATCATCAG
GGTATAGGGTTGTTTTAATACTAAGTAGATTAAGGCCGCAATATGTCTTTGCGCATTCGCGCAAATCGGATCAAC
CACCGCTCCTGGGCATGGTGGCTCTGGCGATAGCTTTACCTTCTCCTAGCGTGCATGAAATTCGCTTAGAATCG
GATATGTTCGTCACTAGGATAAACTTCGACTTTAAAATAGCTCATTGCGAACCGAAAGTGATAGAGTTGCTAGATT
ATTCTCCTGAAGAACTGACTGGCAGGAACCTGTACGCTTTGTGCCTTGGCGAAGATGCTAATAAGCTTAGGAAAA
GTCATATAGACTTAATAAATAAGGGTCAAGTACTAACCAACTACTATAGGATAATGAATAAAAATGGCGGCTACAC
ATGGGTTCAAACGTGCGCCACTGTAGTTTGCAACTCCAAAAATGCCGAAGAACAAAATATTATTTGTGTGAATTAT
GTAATAAGTGGAAGAGAATATGAAAATTTAATAATGGACTGTTGTCAAATGGAAGACAATCCTCACATAGTAAAGA

AGGAGGAGGCCAGCAGTAATGACCCAGAAAACGGATCTCCTGATGCAGATCGCGGGGATGACCGAAATAGCGG
AGGACCCCCCAACC

>Seq4 [organism=Euwallacea validus] RPS3 mRNA, partial CDS
ACAGAGATTATTATCATGGCTACCAGAACTGACCGTGTACTGGGTGAAAAGAACAGGAGGATCAGGGAATTAAC
TTCTGTGGTTCAAAAGAGATTCAACTTCCCAGAAAATTCCGTCGTACTATATGGAGAAAAAGTAGCCAATAGGGG
ATTGTGCGCTATTGCCCAAGCTGAATCTCTGAGGTTCAAATTAATTGGAGGTTTGGCGGTTCGAAGAGCGTGTTA
TGGTGTTTTGAGGTACATTATGGAGTGCGGTGCAAAGGGTTGTGAAGTTGTTGTGTCGGGCAAATTGAGAGGAC
AGAGGGCAAAATCCATGAAATTCGTAGATGGGCTCATGATCCACTCTGGCGATCCTTGTAATGAGTATGTCGACA
CCGCCACTAGACATGTATTGCTTAGACAGGGTGTCTTGGGAATCAAGGTCAAAATCATGCTA

>Seq5 [organism=Euwallacea validus] EF1A mRNA, partial sequence
GAGGCACAAGAAATGGGTAAAGGTTCCTTCAAATATGCCTGGGTATTGGATAAATTGAAGGCTGAGCGTGAACG
TGGTATCACCATTGACATTGCCCTATGGAAATTCGAAACTGCAAAGTACTATGTAACCATCATTGATGCCCCTGG
ACACAGAGATTTCATCAAGAACATGATCACTGGAACGTCCCAGGCTGATTGTGCTGTGCTAATTGTAGCTGCTGG
TACTGGTGAATTTGAAGCTGGTATTTCGAAAAATGGACAGACCAGAGAGCATGCTCTTCTCGCCTTCACTCTTGG
AGTAAGACAACTTATTGTCGGTGTCAACAAAATGGATTCCACCGAACCGCCCTATAGCGAGTCTCGATTTGAGGA
AATTAAAAAGGAAGTCTCTTCTTATATCAAGAAGATTGGTTACAATCCGGCTGCTGTCGTTTTTGTACCTATTTCTG
GTTGGCATGGAGATAACATGTTAGAACCATCCAACAAGATGCCATGGTTCAAGGGATGGTCCATTGAACGTAAA
GAAGGAAAGGCAGAAGGCAAGACCCTCATTGATGCTTTGGACGCCATTCTGCCGCCTAGTCGCCCAACTGACAA
ACCTCTTCGTCTGCCATTACAGGATGTCTACAAAATTGGTGGTATTGGAACAGTACCAGTTGGTCGTGTTGAAAC
TGGTGTCCTCAAGCCTGGTATGGTT
